# HTLV-1 and Innate Immunity

**DOI:** 10.3390/v3081374

**Published:** 2011-08-08

**Authors:** Chloé Journo, Renaud Mahieux

**Affiliations:** 1 Retroviral Oncogenesis Laboratory, INSERM-U758 Human Virology, 69364 Lyon cedex 07, France; 2 Ecole Normale Supérieure de Lyon, 69364 Lyon cedex 07, France; 3 IFR 128 Biosciences Lyon-Gerland, 69364 Lyon cedex 07, France

**Keywords:** HTLV-1, innate immunity, interferon, monocytes, dendritic cells, natural killer cells

## Abstract

Innate immunity plays a critical role in the host response to a viral infection. The innate response has two main functions. First, it triggers effector mechanisms that restrict the infection. Second, it primes development of the adaptive response, which completes the elimination of the pathogen or of infected cells. *In vivo*, HTLV-1 infects T lymphocytes that participate in adaptive immunity but also monocytes and dendritic cells that are major players in innate immunity. Herein, we will review the interplay between HTLV-1 and innate immunity. Particular emphasis is put on HTLV-1-induced alteration of type-I interferon (IFN-I) function. *In vitro*, the viral Tax protein plays a significant role in the alteration of IFN synthesis and signaling. Despite this, IFN-I/AZT treatment of Adult T-cell Leukemia/Lymphoma (ATLL) patients leads to complete remission. We will discuss a model in which exogenous IFN-I could act both on the microenvironment of the T-cells to protect them from infection, and also on infected cells when combined with other drugs that lead to Tax down-regulation/degradation.

## 1. Introduction

Upon viral infection, several defense mechanisms cooperate to limit propagation of the pathogen within the organism. Classically, two types of immune responses have been defined: the innate response, a rapidly engaged but transient and poorly specific response, and the adaptive response, a delayed but specific response which allows the development of immune memory.

### 1.1. Innate Immune Response

In 1957, Isaacs and Lindenmann observed that culture medium from influenza-infected cells induced resistance in naive cells. They hypothesized that a substance secreted by the infected cells inhibited the viral spread [1]. The term “interferon” was coined to name this interfering substance, which was later described as a family of conserved cytokines. Among this family, type I interferons (IFN-I, *i.e.*, IFN-α and IFN-β) are highly effective anti-viral mediators. IFN-I production is not constitutive but must be induced when the cell senses an infection.

All cells express viral sensors, either at the cell surface, at the endosomal membrane or in the cytoplasm [2,3]. These receptors recognize structural motifs specifically present in pathogens and termed PAMPs (*Pathogen-Associated Molecular Patterns*). Examples of viral PAMPs include double-stranded RNA, single-stranded RNA, non-methylated CpG DNA and envelope glycoproteins. The sensors that recognize these viral PAMPs, such as TLRs (*Toll-Like Receptors*) and RLHs (*RIG-I-Like Helicases*), are referred to as PRRs (*Pattern Recognition Receptors*). Interaction of the PAMPs with the PRRs triggers signaling pathways that lead to activation of IRF3 (*Interferon Regulatory Factor 3*) and IRF7 transcription factors, eventually allowing transcription from the IFN-I promoters [4,5].

IFN-I displays both autocrine and paracrine effects. Engagement of the IFN-I receptors (IFNAR-1 and IFNAR-2) at the cell surface induces activation of Jak1/Tyk2 and STAT1/STAT2 signaling cascade that subsequently promotes the expression of a large number of anti-viral effectors known as interferon-stimulated genes (ISGs) [6]. As an example, PKR (*dsRNA-dependent Protein Kinase*), a well-studied representative of ISGs, is a serine-threonine kinase that inhibits translation upon viral infection by phosphorylating the initiation factor eIF2α [7]. Active PKR limits viral proteins synthesis, thus hindering viral replication.

IFN-I can be produced by all cells upon sensing virus infection, and in particular by innate immune cells such as macrophages and dendritic cells (DCs). Plasmacytoid dendritic cells (pDCs) are the major IFN-I producers. These CD123^+^ CD4^+^ CD11c^−^ DCs are found in an immature stage in the peripheral blood, and more predominantly in inflamed tissues [8]. They express TLR7 and TLR9, which trigger their maturation upon engagement. Myeloid CD11c^+^ CD4^+^ dendritic cells (myDCs, also known as conventional DCs) represent a distinct TLR1-8 and TLR10-expressing DC type that circulates in the blood and constantly migrates into secondary lymphoid organs [8]. Many *in vitro* studies of DCs use myeloid-like DCs generated from CD14^+^ monocytes. It should however be kept in mind that such *in vitro* monocyte-derived DCs (MDDCs) are not identical to *in vivo* or *ex vivo*-purified DCs. Therefore, those results should sometimes be handled with caution [8].

In addition, other effectors of innate immunity such as restriction factors are present in cells before infection takes place and can act as immediate inhibitors of a given infectious agent [9]. When these proteins are not able to block viral spread, another efficient defense strategy consists of killing the infected cells. Members of both the innate system (natural killer cells or NK cells) and the adaptive system (cytotoxic T lymphocytes or CTLs) have the ability to kill infected cells. NK cells are able to detect abnormal cell surface phenotypes such as the loss of MHC complexes on target cells. Then, they induce lysis of this target. Invariant NKT (iNKT) cells are a small population of T cells that express cell surface antigens associated with the NK lineage. These cells display attributes of both innate and adaptive immunity, and mature iNKT cells modulate the immune response by secreting large amounts of cytokines.

Innate immune cells such as macrophages and DCs belong to the family of professional antigen-presenting cells. They have the capacity to capture antigens, process them and display them to lymphocytes [8]. Antigen presentation is therefore a central process in the immune response, since it allows priming of the adaptive response, thus bridging the innate and adaptive responses. For this reason, beyond the direct IFN-I-mediated anti-viral role described above, innate immunity is also essential for the activation of adaptive immunity.

### 1.2. HTLV-1

It is estimated that the human T-lymphotropic virus type 1 (HTLV-1) retrovirus infects 15 to 20 million individuals throughout the world [10]. The HTLV-1 antibody prevalence rate varies from 0.2 to 10% among adults, depending on the geographical area. It increases with age, in some places eventually reaching 20 to 50% of the female population aged 60 and above [11].

Three modes of transmission are known for HTLV-1. (1) Mother-to-child transmission, the efficiency of which varies from 10 to 20% and which occurs after prolonged breastfeeding, mainly after 6–9 months of age [11,12]. (2) Sexual transmission [13], Japanese studies clearly demonstrated a higher transmission efficiency from male to female than from female to male. Such differences might account for the increased HTLV-1 seroprevalence with age that is observed in females. (3) Intravenous transmission, mainly through blood transfusion which appears to be the most efficient mode for HTLV-1 transmission. The risk of transmission after transfusion of infected blood products was estimated to reach 15 to 60% [14].

The two major diseases associated with HTLV-1 (*i.e.*, Adult T-cell Leukemia/Lymphoma or ATLL and HTLV-1 Associated Myelopathy/Tropical Spastic Paraparesis or HAM/TSP) are described in all endemic areas [10]. ATLL is a CD4^+^ T-cell malignant lymphoproliferation that is characterized by clonal integration of the HTLV-1 provirus in tumor cells [15]. Shimoyama *et al.* have proposed a classification of ATLL into 4 subtypes: smoldering, chronic, lymphoma and leukemic/acute [16]. Both chronic and smoldering types can progress into the acute form.

HAM/TSP is mainly defined as a spastic paraparesis resulting from a chronic immune inflammation [17,18]. HAM/TSP is a slowly progressive disorder: after 10 years, roughly 50% of the patients are limited to a wheelchair. The possible mechanisms that explain how HTLV-1 causes HAM/TSP include (1) a direct toxicity caused by HTLV-1 specific CTLs; (2) autoimmunity; or (3) bystander damage caused by cytokines such as TNF-α [19]. In most cases, the neurological features include spasticity and/or hyper-reflexia of the lower extremities, urinary bladder disturbance and lower extremity muscle weakness [20].

Interestingly, contamination through blood is associated with a specific risk of developing HAM/TSP [21], while early HTLV-1 infection of babies through infected breast-milk appears to be a major risk factor for developing ATLL but not HAM/TSP. Indeed, several studies conducted in Japan as well as in the Caribbean area and in Brazil have demonstrated that most if not all mothers of ATLL patients were infected with HTLV-1 [22,23].

The role of innate immunity in HTLV-1 pathogenesis is not clear. Some reports convincingly demonstrated that HTLV-1-infected cells do not produce IFN-I and that the Tax protein alters IFN-I signaling [24–27]. However, and even if it does not promote cell cycle arrest or cell death *in vitro* [28], the IFN-α/AZT combination is particularly efficient for treating leukemic, smoldering and chronic ATLL patients and significantly improves their survival [29,30]. In addition, IFN-α, when combined with arsenic trioxide promotes cell death *in vitro* through caspase activation, loss of NF-κB activation and Tax degradation. This regimen was also shown to be efficient *in vivo*, both in a murine ATLL model and for treating ATLL patients [30 –37].

The aim of this review is to provide an overview of the current state of knowledge of the interplay between HTLV-1 and innate immunity. Of particular interest will be the following points: (i) Does HTLV-1 infect cells that participate in the innate immune response *in vivo*? (ii) Can the effectors of innate immunity (and in particular IFN-I) prevent spread of the virus? (iii) Does HTLV-1 infection alter the innate immune response and if so, what are the underlying mechanisms?

## 2. HTLV-1 Infects Cells that Play a Major Role in the Innate Immune Response

*In vivo* and irrespective of the clinical status (*i.e.*, in asymptomatic carriers (ACs), ATLL or HAM/TSP patients) the HTLV-1 provirus is predominantly detected in CD4^+^ T lymphocytes [38], even if infection of CD8^+^ T lymphocytes [39–41], and to a lesser extent of B lymphocytes has also been documented [42]. It thus appears that the main *in vivo* cellular targets of HTLV-1 are cells from the adaptive immune system.

However, innate immune cells (monocytes, macrophages, DCs) are permissive to the virus *in vitro* and/or are infected *in vivo* [39,43–46] (Table 1). As an example, *in vivo* infection of DCs was demonstrated almost 20 years ago in HTLV-1-infected individuals [45]. However, in the absence of any DC-specific antibody available at that time, characterization of the cell subtype remained incomplete. More recently, specific infection of myDCs and pDCs was shown *in vivo* [46]. Although myDCs and pDCs represent less than 1% of the cells in the peripheral blood, thus not significantly contributing to the total proviral load (PVL), their infection could greatly affect immune system function. Of note, the PVL per 10^4^ cells was shown to be proportionally higher in pDCs purified from an HTLV-1 AC than in total peripheral blood mononuclear cells (PBMCs) from the same individual [46], while this was not the case in HAM/TSP patients [47].

How do DCs get infected *in vivo*? Do they represent a dead-end product or do they play a role in the viral transmission? Until recently, the paradigm for HTLV-1 infection was a model in which viral transmission required cell-to-cell contact. In 2008, Jones *et al.* reported that both myDCs and pDCs could be productively infected *in vitro* by cell-free HTLV virions [48]. These DCs could then transmit the virus to T lymphocytes. This led to the establishment of a model in which DCs that are present at the site of infection could be the primary target cells in a newly infected individual, allowing subsequent cell-to-cell transmission of the virus to T cells. Whether these DCs are infected through cell-to-cell contact or by cell-free virus *in vivo* remains to be investigated. However, it is worth noting that although present in the plasma [49], most of the HTLV-1 virus remains cell-associated *in vivo*.

A critical role was assigned to DC-SIGN (*DC-Specific Intercellular adhesion molecule-3-Grabbing Non-integrin*), both for the infection of DCs and for transmission of the virus to T cells [50]. DC-SIGN is a type II transmembrane C-type lectin receptor, whose expression is mostly restricted to myDCs [51]. DC-SIGN contains a carbohydrate recognition domain that is involved in binding to the HTLV-1 envelope and thus mediates HTLV-1 internalization. Furthermore, DC-SIGN binds ICAM-3 that is expressed on T-lymphocytes and mediates transient adhesion of DCs to T cells, possibly allowing transfer of the virus. It was also recently shown that two HTLV-1 viral proteins (p12 and p30) are necessary for the productive infection of monocyte-derived DCs as measured by the presence of p19^gag^ viral protein in the culture supernatant 14 days after viral exposure [52]. Interestingly, macaques exposed to p12 knock out viruses remained seronegative. The exact underlying function of p12 *in vivo* has not been elucidated, but these results suggest that infection of DCs is required to establish and maintain HTLV-1 infection in this animal model.

Monocytes and macrophages might also represent a putative reservoir *in vivo.* Koyanagi *et al.* showed that the PVL ranged between 0 and 140 copies per 10^4^ monocytes in a group of 22 HTLV-1-infected individuals [39]. A latent infection of monocytes, which would allow them to escape immune recognition, was hypothesized [53]. Viral reactivation could then be induced upon monocyte-to-macrophage differentiation. This model is consistent with *in vitro* experiments showing that HTLV-1-LTR-driven *luciferase* transcription was activated upon differentiation of a transiently transfected monocytic cell line into macrophages, in an AP-1-dependent manner [53]. Infection of monocytes and monocyte-derived cells could be of particular interest in the context of mother-to-child transmission. Indeed, breast milk predominantly contains macrophages, rather than T cells. This could explain how prolonged breastfeeding leads to viral transmission. A recent article showed that an *in vitro*-infected breast milk macrophage cell line could transmit the virus to peripheral blood lymphocytes [54], supporting the hypothesis that these cells may be a viral reservoir. However, the presence of HTLV-1 in breast milk macrophages has not yet been reported *in vivo*.

Finally, HTLV-1 infection of activated NK cells was documented *in vitro* [55], but has not been demonstrated *in vivo*. Infection of iNKT cells was however recently demonstrated *in vivo*, in ACs and HAM/TSP patients [47]. The physiopathological consequences of putative NK infection and of iNKT infection will be analyzed in further sections.

## 3. Do Innate Immune Cells Control HTLV-1 Replication?

### 3.1. DCs

**Figure 1.**
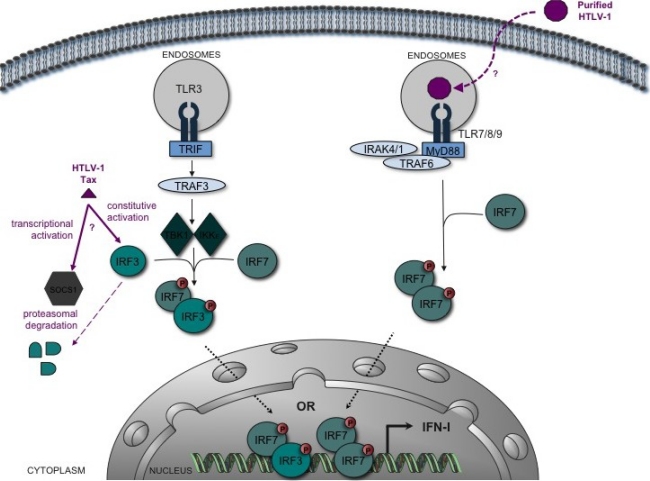
Interplay between HTLV-1 and the type-I interferon (IFN-I) induction pathway. A simplified schematic of the TLR-induced IFN-I induction pathway is shown. Recognition of PAMPs by TLR3 induces the recruitment of the adaptor molecule TRIF. TRIF activates via TRAF3 the kinases TBK1 and IKKɛ that phosphorylate IFR3 and IRF7 transcription factors. IRF3/7 heterodimers activate transcription of IFN-I (especially IFN-β). This initial transcription and synthesis wave is followed by a second wave of IFN-α resulting from an amplification loop that involves IRF7. TLR7, 8 and 9, specifically expressed in pDCs, activate IRF7, which stimulates synthesis of IFN-α via MyD88, IRAK1/4 and TRAF6. *In vitro*, sensing of HTLV-1 induces TLR7-dependent IFN-α secretion by pDCs [59]. However, pDCs isolated from HTLV-1-infected individuals show an altered ability to produce IFN-α in response to viral stimulation (e.g., by HSV-1) [46]. *In vitro*, HTLV-1 Tax targets IRF3 function, but the data are conflicting: while some indicate an inhibitory effect of Tax mediated by SOCS1-induced IRF3 degradation [67], others show constitutive activation of IRF3 by Tax [68].

Even though a number of studies have tried to evaluate whether exogenous IFN-α/β, alone or in combination with other molecules (see below), could play an antiviral role during the treatment of HAM/TSP or ATLL patients, IFN-α production was not measured *in vivo* in HTLV-1-infected individuals (humans or animals). A recent *in vitro* study suggested that normal human pDCs are activated and able to secrete IFN-α by a TLR-7-dependent mechanism when put in contact with concentrated purified HTLV-1 virions [59] (Figure 1). Whether or not those pDCs were infected after exposure to virus was not evaluated [59], although infection of pDCs following exposure to cell-free virus was previously shown by others [48]. Incubation of these pDCs with the virus also led to TRAIL relocalization at the membrane, allowing induction of apoptosis in DR5-expressing T cells. It is therefore possible that HTLV-1 activates pDCs and triggers their differentiation into killer pDCs. Another study showed that a chimeric HTLV-1 (HTLV-1 bearing a MLV envelope) induces the activation of murine bone marrow-derived DCs, as monitored by the up-regulation of surface markers (CD80, CD86 and MHC-II) and the production of IFN-I [58]. These results suggested that infected human pDCs or murine DCs have an intact IFN-I induction pathway.

Hishizawa *et al.*, however, demonstrated that pDCs isolated from a small number of HTLV-1 ACs had impaired IFN-α production, consistent with previous observations reporting impaired immune function in some ACs [64–66]. Is it possible to reconcile these observations? In fact, there is a major difference between those studies. In the first ones [58,59], incoming viruses were used to activate the TLR-7 pathway and subsequently, IFN-I production. In the *ex vivo* study [46], pDCs are likely to be already infected, probably expressing viral proteins, including Tax, which was shown to blunt IFN induction (see below). It is therefore not surprising that those DCs do not produce IFN-I upon re-stimulation.

Finally, Hishizawa *et al.* also found a negative correlation (Table 1) between the PVL in ACs and the ability of pDCs isolated from these individuals to secrete IFN-α *ex vivo* [46]. Although further investigation is needed to demonstrate the causative relationship between both parameters, these results may indicate that efficient IFN-I production could limit viral replication and hence protect against a high PVL. Alternatively, another interpretation of this study is that when PVL increases, the number of infected pDCs also increases and therefore their ability to secrete IFN-I is lowered.

### 3.2. NK Cells

It was suggested that an NK response can be mounted against HTLV-infected cells (Table 1). Indeed, there is a correlation between the level of viral *mRNA* and protein expression in HTLV-1-positive cell lines and their sensitivity to NK-mediated cell lysis [60], suggesting that expression of HTLV-1 triggers alterations of the cell markers that are recognized by NK cells. Similarly, the sensitivity of HTLV-1-positive cell lines to NK-mediated cell lysis was inversely correlated with tumorigenicity in a SCID model [60], implying that NK cells may prevent tumor induction and/or its development *in vivo*. However, whether NK cells represent an efficient defense mechanism is still debated. In addition, results obtained using *in vitro*-infected cell lines were not confirmed when using *in vitro*-infected primary CD4^+^ T cells. A recent study showed that *in vitro*-infected CD4^+^ T cells were susceptible to autologous NK cell lysis similar to mock-infected cells, despite having reduced MHC-I expression [69]. Since viral proteins such as Tax and p12 also affect the expression of markers at the surface of an infected cell (see below), these conflicting results might reflect major differences in viral protein expression levels between *in vitro*-infected cell lines and primary cells. Determining the susceptibility of cells from HTLV-1-infected individuals to NK-mediated lysis should allow a better understanding of the importance of these cells as a defense mechanism against HTLV-1.

### 3.3. iNKT Cells

An inverse correlation between PVL and the frequency of iNKT cells was demonstrated in ACs and HAM/TSP patients [47]. These results suggest that these cells might mount an effective anti-HTLV response and that depletion of this compartment might be involved in HTLV-1 physiopathology. In line with this hypothesis, stimulation of PBMCs isolated from ACs with α-galactosylceramide, the prototypical antigen used for iNKT stimulation, induced *ex vivo* expansion of iNKT cells as well as decreased PVL in the total PBMCs, indicating that expanded iNKT cells have anti-HTLV-1 activity [47].

## 4. Does HTLV Alter the Innate Response?

### 4.1. Alteration of Innate Immune Cell Phenotype, Function and Abundance in the Context of HTLV-1 Infection

Several studies were designed to monitor a possible functional alteration of innate immune cells, in the context of HTLV-1 infection. In a physiological situation, these cells are fully efficient if they are able to arise from precursors in an adequate environment, become activated upon stimulation and differentiate into effectors cells.

#### 4.1.1. Monocytes, Macrophages and DCs

A series of studies performed in the 90s had shown that HTLV-1-infected macrophages and DCs could favor T cell proliferation [45,70,71]. This suggested that infection enhanced the antigen-presentation and T cell-stimulation functions of DCs, potentially participating in the spontaneous T cell proliferation that has been observed in patients. However, this model was not supported in recent results, which demonstrated that monocytes isolated either from HTLV-1-infected ACs, or individuals with ATLL or HAM/TSP, are deficient in their ability to differentiate into functional DCs [56,57]. In a physiological context, activated DCs express high levels of adhesion molecules, MHC antigens and co-stimulatory molecules, which all cooperate to potentiate the antigen-presenting function of these cells, and their ability to induce activation of T lymphocytes. In addition, DCs derived from *in vitro* cultured infected monocytes express abnormal levels of CD1a, CD83, CD86 and HLA-DR, and are poorly able to activate autologous T lymphocytes. As mentioned earlier, MDDCs are not identical to *in vivo* or *ex vivo* purified cells [8]. Hence, the *in vivo* relevance of these results should be carefully investigated.

pDCs are the main producers of IFN-I. As stated above, isolation of pDCs from HTLV-1-infected or uninfected individuals revealed that HTLV-1 status was associated with a defect in IFN-α production after *in vitro* stimulation [46]. The impact of HTLV-1 infection on the number of DCs was also assessed *in vivo* [46,47]. ATLL and HAM/TSP patients had a lower absolute number of myDCs and pDCs than uninfected individuals, while this was not the case for ACs. The correlation between DC number and clinical status suggests that the depletion of DCs could play a role in HTLV-1 physiopathology. It should however be emphasized that measurements of cell numbers were performed on peripheral blood samples. Whether DCs present in the blood accurately reflect the systemic pool of DCs that are present in other tissues is currently unknown. In particular, the observed depletion of DCs in the blood could be the consequence of a massive migration of DCs into tissues.

#### 4.1.2. NK Cells

Similar to monocytes and DCs, altered function and frequency of NK cells were reported in HTLV-1-infected individuals. Significantly decreased NK cell activity [72], together with decreased NK cell abundance was described in HAM/TSP patients, [61]. A low frequency of NK cells was reported in HAM/TSP as well as in ATLL patients [47]. However, NK cells from ACs and from patients with HAM/TSP showed a high proliferation rate *ex vivo*, that was similar to the high proliferation rate of CD8^+^ T cells in infected individuals [62]. Since NK proliferation was measured without prior cell sorting, it may be due to paracrine interactions among PBMCs, rather than to viral expression, which has not been demonstrated in NK cells so far. This ability to proliferate *in vitro* might not be visible *in vivo* due to competition for limited resources with other cell types such as infected CD8^+^ T cells that are also known to have a high proliferation rate. Moreover, the absolute number of NK cells for each infected individual (rather than their frequency among total PBMCs) was not provided in this study. It is therefore possible that the number of NK cells present in the blood might not reflect the total number in the whole body [47]. Thus, the effect of HTLV infection on the physiology of NK cells still needs to be clarified.

#### 4.1.3. iNK T cells

iNKT cells were shown to be less abundant in HAM/TSP and ATLL patients than in uninfected individuals [47,63]. iNKT cells from infected individuals also demonstrated a low proliferation rate and reduced levels of perforin production in response to *ex vivo* stimulation, indicating a severe functional impairment [47].

Taken together, these results indicate that HTLV-1 can alter innate immune cell functions, which has led some groups to search for a role of individual viral proteins in modulation of the molecular pathways involved in innate function.

### 4.2. Alteration of Molecular Pathways by HTLV-1

#### 4.2.1. Modulation of IFN-I Production

IRF3 and IRF7 play essential roles in the early phase of IFN-I gene activation. IRF3 is constitutively expressed and activated through its carboxy-terminal phosphorylation by IKKɛ and TBK1. This promotes the transactivation of downstream genes such as IFN-β. In contrast, IRF7 protein is synthesized *de novo* upon IFN stimulation and contributes to amplification of the IFN response, by inducing expression of IFN-α [67]. A very recent study of *ex vivo* CD4^+^ cells isolated from 30 HTLV-1-infected individuals (ACs, HAM/TSP, ATLL) demonstrated that SOCS1 (*Suppressor Of Cytokine Signaling 1*) is strongly up-regulated in ACs and patients with HAM/TSP but not in those with ATLL. This protein was previously shown to suppress IFN signaling by preventing STAT1 phosphorylation [73]. Interestingly, *SOCS1* expression correlated with HTLV-1 PVL in CD4^+^ cells obtained from HAM/TSP patients. Tax was recently shown to promote the expression of SOCS1 *in vitro* [74]. In PBMCs transfected with an HTLV-1 molecular clone, IRF3 dimer formation (a marker of its activation) does not occur, and SOCS1 expression seems to induce its proteasomal degradation (Figure 1). In contrast, silencing IRF3 expression in HTLV-1 transfected Jurkat cells resulted in increased HTLV-1 mRNA expression. Together, these results suggest that HTLV-1-infected cells that express viral mRNAs (and in particular *tax*) are likely to be impaired for early IFN induction signaling.

These observations are, however, not consistent with a previous report where Suzuki *et al.* showed that HTLV-1 infection and in particular Tax expression leads to constitutive IRF3 phosphorylation and activation in HTLV-1-transformed cell lines [68] (Figure 1). It is worth noting that these studies were not performed using the same experimental models: primary cells transfected with a viral DNA molecular clone (most likely expressing low levels of virus) [67] *vs.* laboratory cell lines where 100% of the cells are infected and constitutively produce high amounts of infectious virions [68].

Additional experiments should therefore be performed to determine the effect of HTLV-1 infection on IRF3 levels or activity, and conversely, the effect of IRF3 activity on HTLV-1 infection. For example, it would be of interest to transfect primary cells with an HTLV-1 molecular clone, then co-cultivate them with an HTLV-1 reporter cell line where IRF3 expression would be suppressed. This would reveal whether or not IRF3 plays a role in HTLV-1 expression following virus infection. One could also measure levels of IFN secretion in cells that overexpress SOCS1.

The viral p30 protein was also shown to modulate innate cell activation [75]. Expression of p30 in human macrophages (*i.e.*, the THP-1 cell line) alters TLR4 signaling, a critical pathway in the innate response to bacterial infection, and inhibits the production of pro-inflammatory cytokines normally secreted in response to TLR4 stimulation [75]. This altered TLR4 signaling is due to down-regulation of TLR4 expression and is mediated by an interaction-dependent inhibition of the transcriptional factor PU.1. p30-mediated inhibition of pro-inflammatory signaling is accompanied by a stimulation of anti-inflammatory cytokine secretion such as IL-10, suggesting that p30 might interfere with the balance of pro- and anti-inflammatory responses during bacterial infection.

#### 4.2.2. Modulation of IFN-I Signaling

**Figure 2.**
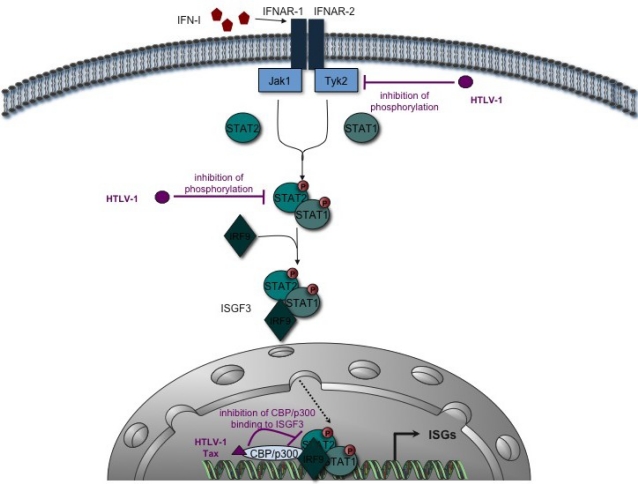
Alteration of the IFN-I signaling pathway by HTLV-1. Binding of IFN-I on IFNAR-1 and IFNAR-2 IFNAR receptors activates the Jak1 and Tyk2 kinases, which phosphorylate STAT1 and STAT2 proteins. Activated STATs form heterodimers that bind to IRF9 to generate the ISGF3 complex. This complex migrates into the nucleus and activates the transcription of ISGs by recruiting the co-activator CBP/p300. HTLV-1 hinders Tyk2 and STAT2 phosphorylation. Moreover, HTLV-1 Tax interacts with CBP/p300 and inhibits its recruitment by the ISGF3 complex. From data published in [25,27].

Following IFNAR-1/IFNAR-2 engagement by IFN-I, the Jak1 and Tyk2 proteins are phosphorylated and induce a signaling cascade involving STAT1 and STAT2. Several reports showed that HTLV-1-infected cells still express IFNAR receptors [25,26]. Therefore, if a defect in IFN signaling exists, it must be downstream from IFNAR binding. Indeed, one report has shown that HTLV-1 prevents IFN-I signaling in HeLa cells *in vitro* [25] (Figure 2). Interestingly, HTLV-1 expression led to decreased Tyk2 and STAT2 phosphorylation, two major players in the IFN-I signaling pathways. This effect was not mediated through *env* or *pol* expression, was independent of the NF-κB pathway, and might be mediated through Gag or Protease expression. Unfortunately, the effect of Tax overexpression was not analyzed, so it remains unclear whether or not this protein is involved in the process. Downstream of Tyk2 and STAT2 phosphorylation, the active ISGF3 complex (*Interferon Stimulated Gene Factor* 3), containing phosphorylated STAT1, STAT2, IRF9, and the CBP/p300 transcription co-activators, triggers the expression of a number of genes. In addition to its effect on SOCS1, Tax overexpression also alters ISGF3 function by preventing interaction of the STAT2 component of ISGF3 with CBP/p300, therefore leading to modulation of the IFN-α transduction cascade [27] (Figure 2). The level of STAT2 phosphorylation was however not determined in this work.

#### 4.2.3. Modulation of Cell Susceptibility to NK-Mediated Cell Lysis

As mentioned above, whether NK cells can lyse HTLV-1-infected cells is not clear. It has been established that the viral auxiliary protein p12, an endoplasmic reticulum-resident protein, can interact with the MHC-I heavy chain and inhibit its maturation [76]. This leads to the retro-translocation of MHC-I out of the endoplasmic reticulum and its degradation by the proteasome, thus limiting its expression at the cell surface [76]. While this allows escape from CTL recognition, it should also allow NK cells to recognize infected cells. Interestingly, it was shown that HTLV-1-infected primary CD4^+^ T cells have a p12-dependent decreased expression of ICAM-1 and -2 adhesion molecules, and of NK cell activating receptor NCR and NKG2D ligands, which protects them from NK-mediated lysis [69]. Thus, p12 expression might confer resistance to both adaptive and innate NK-dependent cytotoxicity mechanisms.

## 5. Treating HTLV-1 ATLL or HAM/TSP Patients with IFN-I

IFN-I has cytostatic and antiviral properties and therefore provides a possible approach for treating chronically infected individuals. Though the literature suggests that (i) HTLV-1-infected cells have an impaired ability to activate innate immunity and that (ii) a number of HTLV-1 encoded proteins blunt IFN-I signaling, a series of clinical trials using IFN-I were performed in Japan [77–83] and in the United States [84] to treat HAM/TSP patients with IFN-α or -β. While some of these studies only analyzed clinical parameters [77], others also evaluated virological markers [82,84]. Doses ranged from 0.3 to 3.10^6^ (IU) by intra-muscular injection for a period ranging from 4 to 793 days. However, in most cases, the treatment duration did not exceed a month. IFN-α treatment of a group of 25 HAM/TSP patients led to a significant although slight decrease of HTLV-1 PVL and to a limited improvement of the clinical status [82]. This study also demonstrated that IFN-α treatment was more efficient in patients with shorter disease duration and a better OMSD (Osame’s motor disability) score. Interestingly, it was also reported that the frequency of the CD8^+^ effector T cells inversely correlated with PVL [82]. Nevertheless, both PVL and clinical symptoms returned to pretreatment values few weeks/months after drug administration was stopped.

Oh *et al.* evaluated the clinical and virological efficacy of escalating doses of IFN-β (30 μg once a week up to 60 μg twice a week) during a 28-week period in a small number of HAM/TSP patients (n = 12) whose disease duration ranged from 2 to 20 years [84]. Although the clinical benefits (improvement in the Nine-Hole Peg test) were very limited, and even if the PVL was not affected by treatment, a reduction of *tax* mRNA and of HTLV-1 specific CD8^+^ cells was observed.

IFN-α combined with AZT is now being used to treat the leukemic, chronic and smoldering forms of ATLL [29], although its precise mechanism of action remains to be determined [85]. IFN-α was also recently used in combination with AZT and arsenic trioxide in a phase II study that was performed in 10 newly diagnosed patients with chronic ATLL, and showed some promising effects with 100% survival, no relapse and no progression after several months [34,35].

## 6. *In vitro* IFN-α or -β Treatment of HTLV-1-Infected Cells

After it was shown that IFN-I displayed some effects *in vivo* (almost exclusively when combined with other drugs), researchers tried to define its mechanism of action *in vitro*. One study demonstrated that treating 293T cells transfected with an HTLV-1 molecular clone (*i.e.*, DNA) with IFN-α led to decreased virion production measured by the presence of p19^Gag^ in the cell culture supernatant. This decreased virion production was not a result of reduced transcription or translation but rather, was due to inhibition of HTLV-1 assembly by preventing the interaction of p19^Gag^ with lipid rafts [24]. The recent discovery of tetherin as a new component of the innate immune response that is inducible by IFN [86] and prevents HTLV-1 release [87] might explain these results, and should be investigated.

Consistent with this work, Kinpara *et al.* then reported that co-cultivation of 293T human cells or NIH3T3 murine cells together with IL-2-dependent HTLV-1-infected cells derived from different ATLL patients also resulted in decreased p19^Gag^ in the cell culture supernatant. However, in this study the effect was due to transcriptional repression of HTLV-1 expression, and these effects were mediated mostly through IRF7-dependent IFN-β secretion by murine epithelial cells [88]. Interestingly IFN-β did not prevent HTLV-1-infected cells from proliferating and did not induce cell death [26,88]. Since lymphoid tissues are enriched in stromal cells *in vivo*, it is conceivable that those uninfected cells would secrete IFN-I after they establish contact with infected CD4^+^ cells. This could control HTLV-1 expression in these infected lymphocytes. Together, these results suggest that, IFN-I can alter HTLV-1 production *in vitro*, although the precise mechanism of action remains to be clarified.

## 7. Conclusions

The different reports that have been published on the interplay between HTLV-1 infection or HTLV-1 protein expression and innate immune cells *in vitro* sometimes seem difficult to reconcile with observations that were made *in vivo*. Indeed, a significant number of reports clearly demonstrate that HTLV-1-infected cells have a reduced ability to act as members of the innate immunity network and that HTLV-1 proteins, in particular Tax, prevent both type I IFN secretion and IFN signaling. Nevertheless, combinations of IFN-α/AZT or IFN-α/AZT/arsenic trioxide represent efficient means to obtain complete remission in most ATLL subtypes (except the lymphoma type) and in the murine ATLL model. This might be due to the fact that human or murine ATLL cells barely express Tax and, therefore still possess an intact IFN signaling pathway and are sensitive to IFN-α/AZT. In addition, arsenic trioxide, by promoting the degradation of Tax could also restore IFN signaling. Less pronounced IFN-α effects in HAM/TSP patients are likely to be due to a higher Tax expression, which then impairs IFN signaling. Finally, in ATLL patients exogenous IFN-I might also act on uninfected cells and render them less susceptible to infection.

In conclusion, understanding the intimate interplay between HTLV-1 and the innate immune response will require additional studies. It will also be crucial that future work aimed at defining these interactions be performed with patient samples and primary cells in order to avoid overexpression systems that might lead to misinterpretation of the results.

## Figures and Tables

**Table 1.** Interplay between innate immune cells and HTLV-1. See text for details. ND, not determined.

		**Infection by HTLV-1**	**Control of HTLV-1 Replication**	**Infection-Induced Alteration of Cell Numbers *in vivo***	**Infection-Induced Alteration of Function**
**Monocytes / Macrophages**		Demonstrated *in vitro* and *in vivo* [39,43,44]	ND	ND	Alteration of the *in vitro* differentiation into functional dendritic cells (DCs) [56,57]
**Dendritic Cells**	myDCs	Demonstrated *in vitro* and *in vivo* [45]^1^ [46,48]	- Secretion of IFN-α upon *in vitro* HTLV-1 sensing [58]^2^	Decreased (ATLL and HAM/TSP patients) [46,47]	ND
	pDCs	Demonstrated *in vitro* and *in vivo* [46–48]	- Secretion of IFN-α and maturation into killer pDCs upon *in vitro* HTLV-1 sensing [59] - Inverse correlation between the PVL and the ability of *ex vivo*-stimulated cells to produce IFN-α (ACs) [46]	Decreased (ATLL and HAM/TSP patients) [46,47]	Alteration of the ability to secrete IFN-α upon *ex vivo* stimulation (ACs) [46]
**Natural Killer Cells**		Demonstrated only *in vitro*, on activated cells [55]	Inverse correlation between infected cell lines sensitivity to NK-mediated cell lysis and tumorigenicity in SCID mice [60]	Decreased (ATLL and HAM/TSP patients) [47,61]	High rate of *ex vivo* proliferation (ACs and HAM/TSP patients) [62]
**Invariant Natural Killer T Cells**		Demonstrated *in vivo* [47]	Inverse correlation between the PVL and iNKT cells frequency (ATLL and HAM/TSP patients) [47]	Decreased (ATLL and HAM/TSP patients) [47,63]	Low rate of *ex vivo* proliferation and perforin production in infected individuals [47]

1 Incomplete cell subtype characterization in [45] due to the absence of any DC-specific antibody available at that time;

2 Murine bone marrow-derived DCs.
